# Supplementary material for: MetaRibo-Seq measures translation in microbiomes
Source: Nat Commun. 2020 Jun 29;11:3268. doi: 10.1038/s41467-020-17081-z (PMC7324362; doi:10.1038/s41467-020-17081-z)
Supplement: Supplementary file 10 — Supplementary Data 7 [file 41467_2020_17081_MOESM10_ESM.zip › File2/Confidence_High_Taxonomy/155127_out.krona.html]

Javascript must be enabled to view this page.

members
magnitude
magnitudeUnassigned
count
unassigned
taxon
rank

155127\_out

106

superkingdom
2
106

106
976
phylum

106
200643
class

106
order
171549

family
815
106

106
genus
816

1
457392
species

SRS147557\_contig\_number\_29582

1
46506
species

SRS098644\_contig\_number\_11636


SRS018541\_contig\_number\_5477
820
species
1

100
species
817

SRS011084\_contig\_number\_256SRS012902\_contig\_number\_139SRS013158\_contig\_number\_55SRS013215\_contig\_number\_925SRS013476\_contig\_number\_32447SRS014459\_contig\_number\_24641SRS014736\_contig\_number\_2701SRS015065\_contig\_number\_13203SRS015190\_contig\_number\_1091SRS015264\_contig\_number\_13164SRS015578\_contig\_number\_15828SRS015694\_contig\_number\_6417SRS015854\_contig\_number\_18836SRS015890\_contig\_number\_1308SRS015960\_contig\_number\_21465SRS016095\_contig\_number\_1867SRS016132\_contig\_number\_4371SRS016267\_contig\_number\_3178SRS016495\_contig\_number\_10916SRS017433\_contig\_number\_215SRS017521\_contig\_number\_15198SRS017622\_contig\_number\_94SRS018313\_contig\_number\_1607SRS018656\_contig\_number\_884SRS018817\_contig\_number\_3806SRS018888\_contig\_number\_4951SRS018984\_contig\_number\_5888SRS019267\_contig\_number\_264SRS019397\_contig\_number\_306SRS019601\_contig\_number\_6874SRS019787\_contig\_number\_13241SRS020622\_contig\_number\_contig-100\_671.671SRS020869\_contig\_number\_27749SRS021484\_contig\_number\_contig-100\_466.159460SRS022524\_contig\_number\_7904SRS023346\_contig\_number\_2382SRS023829\_contig\_number\_148SRS023971\_contig\_number\_246SRS024075\_contig\_number\_5316SRS024132\_contig\_number\_30430SRS024549\_contig\_number\_1178SRS043411\_contig\_number\_5107SRS043701\_contig\_number\_9416SRS046502\_contig\_number\_709SRS046712\_contig\_number\_1002SRS048981\_contig\_number\_2174SRS049402\_contig\_number\_646SRS050299\_contig\_number\_10563SRS051610\_contig\_number\_46SRS053214\_contig\_number\_3373SRS053573\_contig\_number\_278SRS055966\_contig\_number\_762SRS056259\_contig\_number\_12668SRS057717\_contig\_number\_1385SRS058723\_contig\_number\_652SRS063040\_contig\_number\_contig-100\_1244.257317SRS063127\_contig\_number\_2657SRS063190\_contig\_number\_9125SRS063985\_contig\_number\_28994SRS074964\_contig\_number\_3909SRS075398\_contig\_number\_1335SRS075773\_contig\_number\_37558SRS076804\_contig\_number\_contig-100\_421.115458SRS077086\_contig\_number\_2243SRS077194\_contig\_number\_872SRS077335\_contig\_number\_2308SRS077589\_contig\_number\_2934SRS077730\_contig\_number\_2615SRS078419\_contig\_number\_16906SRS097920\_contig\_number\_27033SRS098717\_contig\_number\_14840SRS098881\_contig\_number\_23280SRS104036\_contig\_number\_6695SRS1041039\_contig\_number\_13992SRS1041145\_contig\_number\_12747SRS104311\_contig\_number\_1474SRS104485\_contig\_number\_949SRS104636\_contig\_number\_15081SRS1054928\_contig\_number\_1338SRS1055022\_contig\_number\_1470SRS1055043\_contig\_number\_contig-100\_476.154961SRS1055069\_contig\_number\_contig-100\_1695.78128SRS1055099\_contig\_number\_contig-100\_165.68532SRS140492\_contig\_number\_2587SRS140513\_contig\_number\_2379SRS142599\_contig\_number\_14378SRS143342\_contig\_number\_9398SRS143417\_contig\_number\_15069SRS143598\_contig\_number\_12307SRS144362\_contig\_number\_12367SRS144506\_contig\_number\_3922SRS147022\_contig\_number\_542SRS147139\_contig\_number\_7017SRS147272\_contig\_number\_443SRS147377\_contig\_number\_3035SRS147653\_contig\_number\_618SRS148159\_contig\_number\_12446SRS893279\_contig\_number\_4885SRS893342\_contig\_number\_1682SRS971275\_contig\_number\_7197

1
671267
species

SRS024388\_contig\_number\_1183


SRS098655\_contig\_number\_629
species
291644
1

818
species
1

SRS075021\_contig\_number\_572
